# Small Non-Coding RNAs: New Insights in Modulation of Host Immune Response by Intracellular Bacterial Pathogens

**DOI:** 10.3389/fimmu.2016.00431

**Published:** 2016-10-18

**Authors:** Waqas Ahmed, Ke Zheng, Zheng-Fei Liu

**Affiliations:** ^1^State Key Laboratory of Agricultural Microbiology, College of Veterinary Medicine, Huazhong Agricultural University, Wuhan, China

**Keywords:** small RNAs, *Salmonella*, *Mycobacterium*, *Brucella*, innate immunity, adaptive immunity

## Abstract

Pathogenic bacteria possess intricate regulatory networks that temporally control the production of virulence factors and enable the bacteria to survive and proliferate within host cell. Small non-coding RNAs (sRNAs) have been identified as important regulators of gene expression in diverse biological contexts. Recent research has shown bacterial sRNAs involved in growth and development, cell proliferation, differentiation, metabolism, cell signaling, and immune response through regulating protein–protein interactions or *via* their ability to base pair with RNA and DNA. In this review, we provide a brief overview of mechanism of action employed by immune-related sRNAs, their known functions in immunity, and how they can be integrated into regulatory circuits that govern virulence, which will facilitate our understanding of pathogenesis and the development of novel, more effective therapeutic approaches to treat infections caused by intracellular bacterial pathogens.

## Introduction

Precise control of gene expression is an essential feature of the immune system. The immune system depends on a sophisticated gene expression program equipped with an arsenal of strategies to fight against infections, mainly controlled by well-described transcriptional and posttranscriptional mechanisms (1). Cells of the immune system are able to undergo dramatic changes in transcription mechanisms to efficiently organize expression of genes critical to defense. Innate and adaptive immune cell differentiation and activation depends largely on these transcription events (2). Neutrophils, macrophages, and dendritic cells (DCs) exhibit both common and unique sets of toll-like receptors (TLRs), chemokines, and cytokines in innate immune responses. microRNAs (miRNA) and RNA-binding proteins determine the specific region of the gene accessible to transcription factors that ultimately regulate transcription (1).

Small non-coding RNAs (sRNAs) play critical roles in bacterial gene expression and are recognized as key regulators in bacteria. Typically, these RNA regulators range from 50 to 200 nt in length and act on independently expressed targets, often encoded in the intergenic region (3, 4). sRNA controls bacterial gene expression by employing multiple molecular strategies to regulate the expression of gene targets, including binding directly to complementary sequences present in target mRNA molecules (5, 6). sRNAs interact with their specific target to exert both positive and negative effects on gene expression. In positive regulation, sRNAs bind with target mRNA at the 5′-untranslated region (UTR) and alter the secondary structure of the mRNA, to access a ribosome-binding site (RBS) that allows translation (7, 8). In addition, sRNA can bind to the 3′-UTR of target mRNA, ultimately increasing gene expression and stabilizing the transcript (9). Alternatively, sRNAs can also exert inhibitory effects by binding with mRNA 5′-UTR, resulting in decreased stability, degradation of the mRNA, occlusion of the RBS, and inhibition of translation (10). sRNAs are widely identified and their number constantly growing due to their involvement in diverse biological contexts including cell proliferation, development, differentiation, apoptosis, metabolism, stress response and signal transduction (11, 12).

Pathogenic bacteria have to face hostile and changing environments characterized by high concentrations of reactive oxygen and nitrogen species, low pH, and limited nutrient availability that hinder in their replication and infection to succeed (13). Pathogens have evolved a variety of strategies to survive and replicate within eukaryotic cells, establishing mechanisms to manipulate the host-cell machinery for their own benefit (14). After internalization in host cells, pathogenic bacteria modulate their trafficking to avoid lysosomal fusion by occupying a specialized membrane-bound vesicle. Intracellular bacteria are divided in two classes: vacuolar intracellular bacteria, such as *Salmonella, Mycobacterium, Legionella, Brucella*, and *Coxiella*, that survive and replicate either by avoiding vacuole–lysosome fusion or by altering the phagolysosome environment; and cytosolic intracellular bacteria, including *Francisella, Shigella, Listeria, Burkholderia*, and *Rickettsia*, that usually escape to proliferate within the cytosol of host cell (15, 16).

Recent developments in biocomputation have revealed a large number of regulatory sRNAs and have highlighted their potential links to bacterial pathogenesis (17, 18). Bacterial adaptation to intracellular environment niches is efficiently regulated in both time and space. These newly identified sRNAs play an integral part in virulence expression and bacterial stress responses that are ultimately advantageous for pathogens in adaptation and modification of the host-immune response (19). Understanding the mechanisms adopted by sRNAs to control immune cell function and how immune-related cells maintain cell viability and competitiveness in varying environmental niches is critical. However, roles of sRNA regulators in pathogenesis and immune response mechanisms have only begun to be investigated. In this review, we summarize the mechanisms employed by bacterial sRNAs in gene regulation and sRNA-based strategies to counter host immune response mechanisms as well as their implications in the pathogenesis of intracellular bacteria.

## Mechanisms Employed by Bacterial sRNAs for Gene Regulation

**Figure 1:**
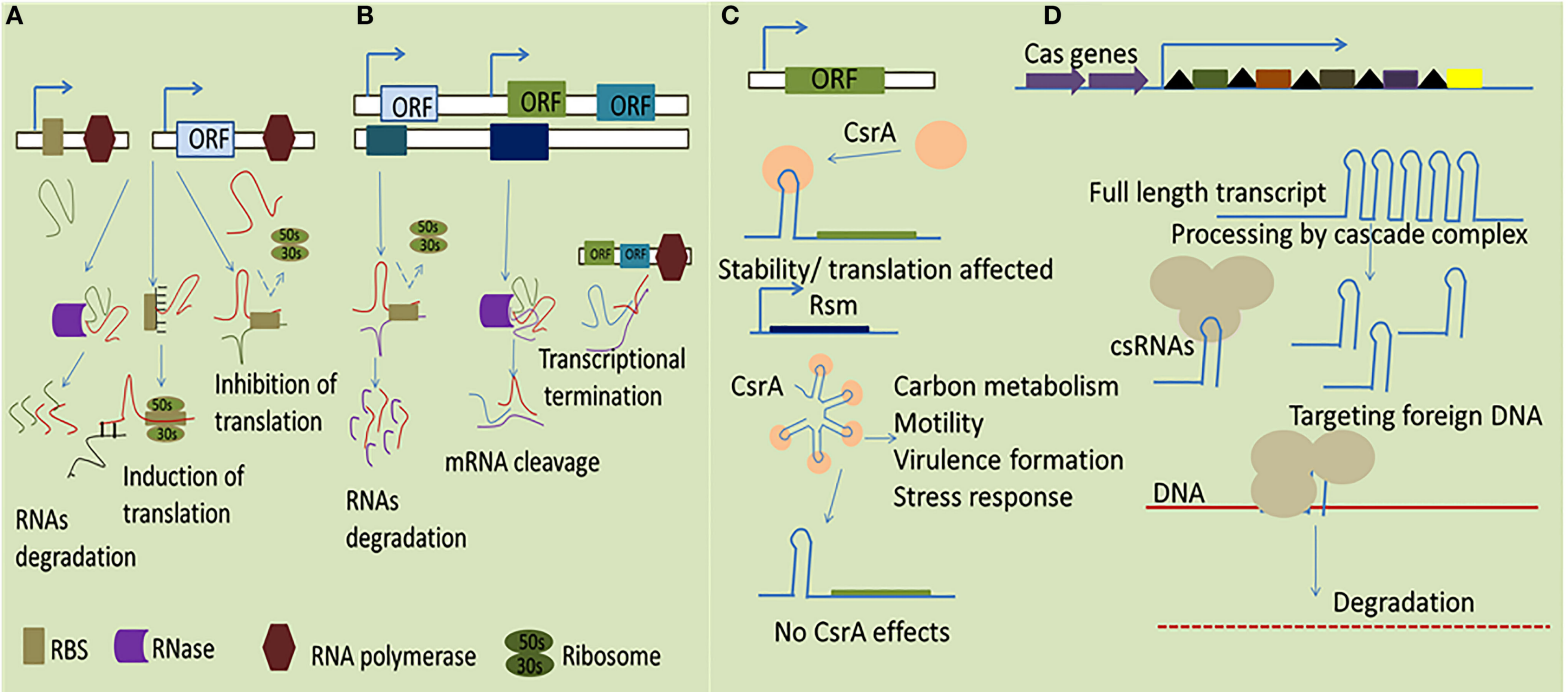
**Simplified representation of mechanisms by which sRNAs function in bacteria**. **(A)** The *trans*-encoded sRNAs interact with their specific target through imperfect base pairing which ultimately results in both positive and negative effect in altering gene expression to promote RNase degradation of the double-stranded RNA molecules. **(B)** The *cis*-encoded sRNAs share extensive complementarity by binding with target mRNA resulting in transcriptional termination, degradation of the sRNA–target RNA complex, and affecting translation through a putative loop formation and, consequently, the cessation of the RNA polymerase activity. **(C)** The dimeric RNA-binding protein CsrA interacts with target mRNA, typically represented in a hairpin loop structure, transcription termination, leading to an alteration of the accessibility of the translation machinery, and/or the stability of the RNA. **(D)** Mechanism of action of CRISPR arrays for transcription full length RNA which directly target the foreign DNA *via* CAS proteins resulting in subsequent degradation of the exogenous DNA.

Bacterial regulatory sRNAs operate at all layers of gene regulation to modulate translation, transcription, DNA maintenance or silencing, and mRNA stability. They use different mechanisms to achieve these outstanding regulatory functions (5). The major mechanisms employed by bacterial sRNAs for gene regulation are as follows:

(i) *Trans*-encoded sRNA are usually encoded on the genome in trans location distinct from their targets and share partial complementarity with their target mRNAs. They potentially establish base pairing to the Shine-Dalgarno (SD) sequence of target mRNAs usually 10–25 nt in length to sequester the RBS (4). Additionally, *trans*-acting sRNAs are firmly coupled with the RNases activity to exploit their regulatory functions resulting in RNA turnover through RNA cleavage (20). In many cases, it is thought that *trans*-encoded RNA molecules engage RNA chaperone Hfq to facilitate sRNA-mediated regulation due to limited complementarity between sRNAs and their mRNA targets (21).
(ii) *Cis*-encoded sRNAs are another class of intracellular bacterial sRNAs complementary to their target encoded in the same region of DNA. They have functional ability to interact autonomously as they are transcribed from DNA strand opposite to genes they regulate (22). *Cis*-encoded sRNAs vary greatly in size and usually located in the UTRs of the corresponding gene to establish firm RNA duplex formation which in turn affects ribosome-binding/translation and rearranges the secondary structures to affect mRNA stability or termination events as shown in Figure 1 (23).
(iii) In addition to RNA–RNA regulation characterized by base pairing, sRNA can also interact with regulatory proteins to directly modify their activities by mimicking and, thus, efficiently compete with DNA or RNA targets. The best suited example for such interaction is CsrA/RsmA family regulators (global carbon storage regulator). All Csr/Rsm-regulatory networks represent a common feature in pathogenic bacteria, the two-component system (TCS) regulate the transcription of the small RNAs to sequester CsrA. The sRNAs directly bind with CsrA/RsmA to sequester it from interacting with mRNA targets usually in close immediate vicinity of the RBS. This phenomena result in the enhancing the translation of the previously blocked transcripts (24).
(iv) Recent studies indicates that a class of sRNAs participates in an adaptive microbial immune system known as clustered regularly interspaced short palindromic repeats (CRISPR), which provide the bacteria with RNA-based acquired immunity against invading DNA elements, such as from bacteriophages, plasmids, and mobile genetic elements (25). This system is composed of an array of conserved short DNA repeat sequences originating from foreign DNA and interspaced by variable spacer regions. Furthermore, together with the conserved Cas proteins, the crRNA can recognize the complementary DNA target to mediate its degradation (26).

## sRNAs as Coordinators of Pathogenesis

After internalization in the host cell, intracellular bacterial pathogens are challenged by diverse changing environmental conditions. Host cells have established mechanisms to counteract the intracellular bacteria, including degradation of pathogens within the lysosomal compartments. Conversely, as successful pathogens, intracellular bacteria have acquired strategies to avoid lysosomal degradation, such as the arrest or delay of vacuolar maturation in *Salmonella* (late endosome) and *Mycobacterium* (early endosome), control of intracellular trafficking in *Brucella* and *Legionella*, and resistance to lysosome action in *Coxiella* (27, 28). These pathogens have developed strategies to evade host protective mechanisms for adaptation, survival, replication, and persistence within host cells to establish chronic infection that is mainly dictated by the presence of certain structural components and virulence factors (29, 30).

The Csr-type system is the most common posttranscriptional regulatory network in intracellular bacteria and is well-characterized in *Legionella* and *Salmonella*. *Legionella* uses effector proteins to modulate host-cell function and establish a replicative niche by forming a membrane-bound vacuole designated the *Legionella*-containing vacuole (LCV) (31). These effector proteins are under the control of CsrA, which participates in the type IVB secretion system (T4SS) to modulate endoplasmic reticulum (ER)–Golgi vesicular trafficking, with involvement of sRNA-binding protein in survival and replication of intracellular pathogens (32). The VipA, RalF, and YlfA effector proteins have been linked directly to vesicular trafficking to alter the host-cell activity (33). The CsrA controls expression of virulence regulatory genes near the Shine-Dalgarno (SD) sequence of target mRNA located on *Salmonella* pathogenicity islands (SPIs). Also the transition from a sessile to a motile life form of *Salmonella enterica* serovar Typhimurium is strongly affected by CsrA (34). In *S. enterica* serovar Typhimurium, CsrA seems to act positively on swarming motility, and it is required for accurate flagella expression by stabilizing the *flhDC* and the *fliA* mRNAs, which are the regulators of the flagella operon. This stabilizing effect leads to an increased production of flagellar proteins (34). In *Legionella*, CsrA also affects the flagella sigma factor FliA, but in contrast to *S. enterica* serovar Typhimurium, overexpression of CsrA in *Legionella pneumophila* resulted in lower *fliA* transcription and subsequently to reduced levels of FlaA, the major structural flagellar protein controlled by FliA (35). Although CsrA is apparently a common regulator for flagella expression in different bacteria, the regulatory function differs significantly between them, as it can be either positive or negative (36, 37). sRNAs, such as RsmY and RsmZ, regulate the expression of effector protein RsmA to affect the replication of *Legionella* in macrophages (38), and directly target T4SS regulatory genes to facilitate intracellular survival of pathogen (39, 40).

**Figure 2:**
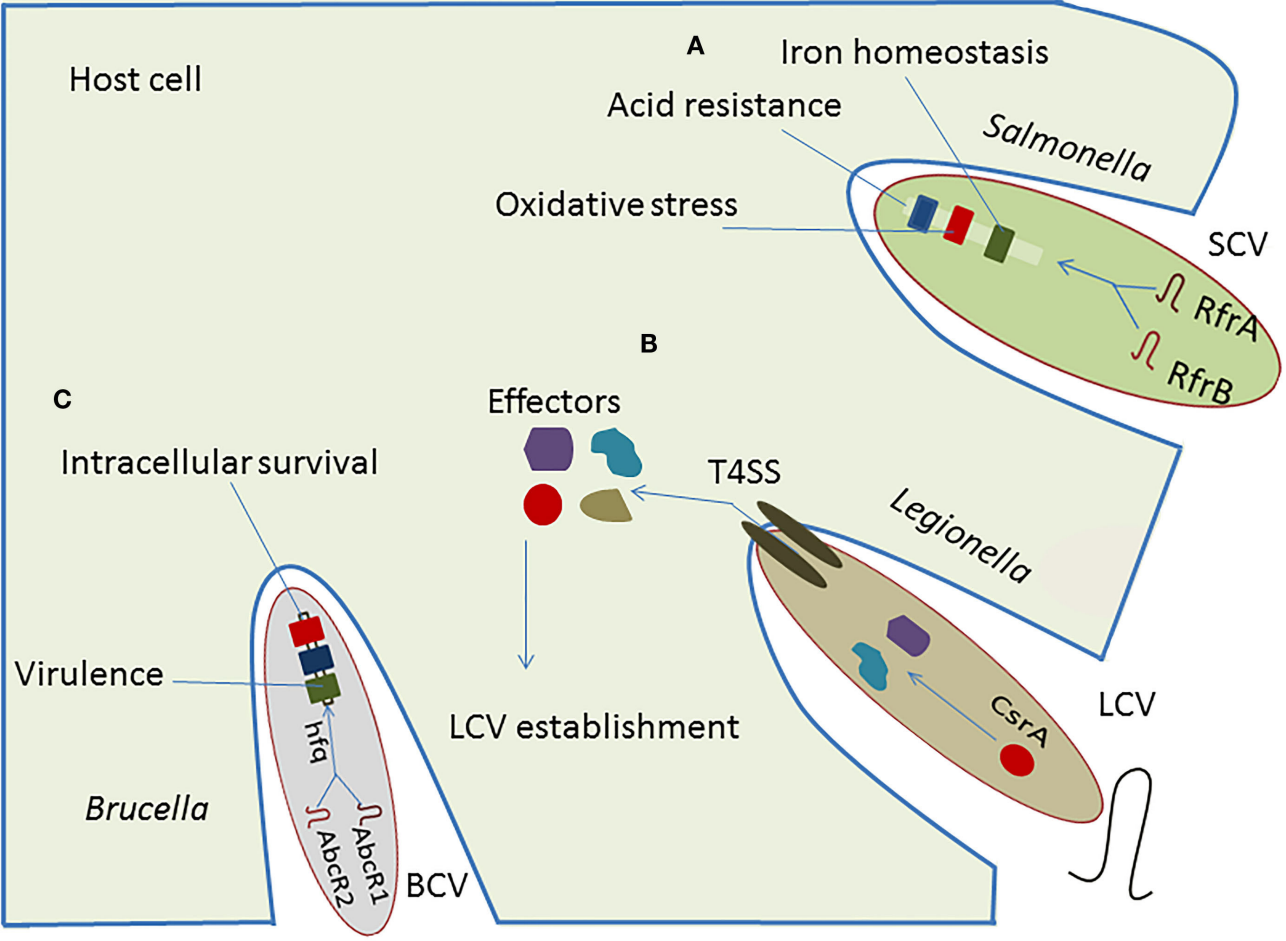
**Implication of RNA-mediated regulation in pathogenesis of intracellular bacteria**. **(A)** The regulatory cascade of *Salmonella* is composed of sRNAs, RfrA and RfrB, plays vital role in the establishment of *Salmonella*-containing vacuole (SCV), which facilitates adoption of pathogen within host cell resulting in regulation of iron homeostasis, oxidative stress, and acid resistance. **(B)** The regulatory sRNAs, RsmY and RsmZ, control the action of effectors secreted in cytosol of host which helps in establishing the *Legionella*-containing vacuole (LCV). **(C)** The regulatory sRNAs, AbcR1 and AbcR2, interact with hfq to modulate the virulence and intracellular survival of *Brucella*.

The RfrA and RfrB are two RybB homologous sRNAs in *Salmonella enterica* that play an essential role in the intracellular replication in macrophages. Additionally, Fur, a well-known repressor of RybB sRNA is also required for phagocytosis and intracellular survival of *Salmonella* in human macrophages (Figure 2) (41). These conserved sRNAs regulate expression of virulence genes involved in the oxidative stress, iron homeostasis, and acid resistance within host cells. Furthermore, they have been induced in THP-1 macrophages, fibroblasts, and murine macrophages, suggesting a complementary role of sRNAs in intracellular replication (42). Caswell et al. (43) reported two homologous sRNA in *Brucella*, named AbcR1 and AbcR2, that play significant role in pathogenicity and establishment of chronic infection. Deletion of AbcR1 or AbcR2 alone in *Brucella* did not affect intracellular growth in macrophages, but deletion of both resulted in its significant attenuation in macrophages, as well as in mouse model of chronic *Brucella* infection (43).

Transcriptional regulators are the subject of considerable study at the molecular level, and the number of newly discovered sRNAs is increasing, with more than 100 identified in *Salmonella* (19). In many cases, the ubiquitous RNA-binding protein, Hfq, establishes dynamic interactions with RNA molecules to function in virulence of intracellular pathogens. Due to limited complementarity between sRNA and target mRNA, Hfq is essential to facilitate RNA–RNA interactions. Indeed, deletion of *hfq* has dramatic impact on virulence and intracellular survival in *Brucella abortus* (44), *L. pneumophila* (45), and *Salmonella typhimurium* (46). In addition, Hfq-bound sRNAs are directly involved in regulating metabolic systems and gene expression such as that required for two-component regulatory systems, lipopolysaccharide biosynthesis, host-cell invasion, fatty acid metabolism, central carbon metabolism, and in motility of bacteria (47, 48).

The sRNA GcvBs regulate the ABC transport system through direct binding with extended C/A-rich regions at the mRNA level resulting in lower expression and inhibition of the ABC transport system in *S. typhimurium* (49). Furthermore, IsrJ sRNA-dependent temporal regulation has been reported in SPIs affecting pathogen invasion of intestinal epithelial cells (50).

IsrM is the SPI-encoding sRNA involved in direct regulation of *HilE* and *SopA* virulence genes, which are key regulators of *Salmonella* virulence and essential for bacteria to evade the host immune system. Specifically, *Salmonella* hinders production of *HilE* and *SopA* for invasion of epithelial cells, which facilitates its survival within the host macrophage (51).

AmgR, a *cis*-encoded 1.2 kb long antisense RNA in *S. typhimurium*, which is complementary to *mgtCBR* mRNA, specifies the MgtC protein. The AmgR sRNA plays an essential role in survival of *Salmonella* within macrophages and in its virulence in mice and is required for replication in a low Mg^2+^ environment (52). Surprisingly, transcription of *mgtCBR* mRNA and AmgR sRNA is controlled by the two-component regulatory system PhoP/PhoQ. In detail, when PhoQ senses decrease level of Mg^2+^ in cytoplasm, it phosphorylates PhoP initiating transcription of *mgtCBR* mRNA by direct binding with *mgtC* and *amgR* promoters. As MgtB and MgtC protein levels are decreased by long RNA regulatory elements, FtsH protease promotes degradation of the MgtR binding to the MgtC protiens. Consequently, AmgR acts as a timing device for sense-encoded MgtC protein, which was shown to diminish virulence in mice (52). Recently, regulatory sRNAs have been identified in *Mycobacterium* that act in pathogenesis by regulating the target gene *Rv0485* that participates in mediation of virulence in mice (53). These studies suggest that intracellular pathogens utilize sRNA-based strategies to establish productive intracellular infection within host cells.

## sRNAs in Modulation of Innate and Adaptive Immune Response

Intracellular pathogens have developed well-organized strategies to cope and interfere with host innate immune mechanisms that ultimately facilitate establishment of an environment favorable for an effective, long-lasting, adaptive immune response (54). The mammalian innate immune response mechanism provides first line of defense against invading bacterial pathogens. The recognition of molecules typical of a microbe pathogen-associated molecular pattern is obtained *via* pattern-recognition receptors (PRR) through TLRs that are expressed at high levels on DC and macrophages. The TLRs transmit signals *via* MyD88 to activate the NF-κB, MAPKs, and IRF signaling pathways to initiate microbial clearance (54). Study of a class of sRNAs designated miRNAs, typically 20–22 nt in length, has greatly expanded our understanding of mechanisms involved in gene expression through posttranscription and translation regulation of protein coding genes. miRNAs play pivotal roles in modulation of innate as well as in adaptive immune response mechanisms (55).

### *Salmonella* 

At the initial stages of infection, innate immune response mechanisms effectively control the replication and survival of *Salmonella*. *In vitro* studies indicate that *Salmonella* modulates miRNAs in both epithelial cells and macrophages. Key host miRNAs, such as miR-155 and miR-146, are upregulated in immune cells in response to intracellular bacterial pathogens, apparently co-induced during physiological processes stimulated by lipopolysaccharides to repress TLR-mediated recognition of bacterial molecules and NF-κB activity (56). Modulation of miR-146a/b, miR-155, and miR-21 were first reported in *Salmonella* infection, with NF-κB-dependent miRNAs significantly induced upon infection in mouse macrophages (57, 58). miRNAs are usually triggered in response to sense extracellular stimulus. This phenomenon was observed in *Salmonella* mutant strains defective in cell invasion (ΔSPI-1) and replication (ΔSPI-2), as well as in human monocytes (58, 59). It was subsequently found that vaccination of miR-155-null mice with attenuated *Salmonella* vaccine did not confer protection, and mice showed severely defective T-cell cytokine production, indicating the complex role of miR-155 in innate immune responses to *Salmonella* (59, 60).

Schulte et al. (56) reported the importance of miR-155 activation by the sensing bacterial peptidoglycan *via* cytoplasmic NOD2 receptor, suggesting its potential role in innate immune response (56). miR-146 function in zebrafish embryos infected with *S. typhimurium* was found to be disrupted by knockdown of the TRAF6-MyD88 pathway that mediates transduction of TLR signals and cytokine activation (61). miR-146 function characterization has revealed that it acts as a negative regulator of IRAK1 and TRAF6 expression, which, in turn, affects NF-κB signaling pathway (62). miRNA let-7 appears to be a factor in the acute innate immune response that participates in the TLR signaling pathway *via* lipopolysaccharide action. In *Salmonella* infection, let-7 was downregulated in a cell-type dependent manner in HeLa and murine macrophage cells, suggesting that repression of this miRNA family constitutes a common signature of the infection of phagocytic and non-phagocytic cells by *Salmonella* (57). Zhang and colleagues reported miR-128 upregulated expression upon *Salmonella* infection that led to reduced secretion of macrophage colony-stimulating factor-mediated macrophage recruitment, in turn suppressing the host immune response mechanisms (63).

Although innate immunity effectively controls replication and survival of *Salmonella* at the initial stages of infection, a well-organized adaptive immune response is also required at later stages of chronic infection (54). *Salmonella* shows the ability to infect phagocytic and non-phagocytic cells, residing inside phagocytic cells in the so-called *Salmonella*-containing vacuole (SCV). *Salmonella* secretes several virulent SPI-2-encoded proteins into the host cytoplasm to facilitate its intracellular replication and invasion into host cells through two distinct type-III secretion systems (T3SS) (64). Mature DCs may have unique tolerogenic properties for initiation and control of adaptive immune responses. *Salmonella* has developed mechanisms to counteract the function of DCs, such as subversion of cellular trafficking by preventing fusion of the SCV with lysosomes, which ultimately facilitates pathogen entry into the host (65). Additionally, MHC-I and MHC-II molecules expressed on the surface of DCs show elevated numbers of bacterial-derived antigens that facilitate activation of T cells, resulting in enhanced host adaptive immune response (66). The B cell intrinsic requirement of miR-155 is essential for IgG1 antibody production in response to thymus-dependent and -independent antigens, following vaccination with attenuated *Salmonella* (67).

Rodriguez et al. reported bic/miR-155-deficient mice show diminished adaptive immune response against *S. typhimurium* after intravenous immunization that failed to establish strong adaptive immunity, likely due to defective antigen presentation as well as impaired B and T cell functioning by DCs (60). In addition to miR-146 and miR-155, miR-21, miR-23b, miR-27a, miR-24, miR-222, and miR-29 showed upregulation upon *Salmonella* infection in human monocytes. The upregulated miRNA shows that monocytes differentiation is involved in modulation of the TGF-β signaling pathway to counteract host defense mechanisms (58).

The miRNA let-7 family members directly target major immunomodulatory cytokines IL-6 and IL-10, and their downregulation results in increased expression of both cytokines in response to *Salmonella* infection (68). miR-21, miR-146a, and miR-155 show strong induction of NF-κB, leading to decreased regulation of B cell and T cell proliferation in murine macrophages upon *Salmonella* infection (69). These findings suggest that *Salmonella* uses miRNA as a strategy to modulate TLR-NF-κB signaling pathways as well as to counteract the function of DCs to subvert its cellular trafficking, and miRNAs play a significant role in the interaction of innate and adaptive immune mechanisms.

### *Mycobacterium* 

Recently, several studies have highlighted the role of miRNAs in mycobacterial infection (70). *Mycobacterium* also modulates miRNAs associated with signaling pathways, which enhances its survival in the host. miR-155 was found to be upregulated in *Mycobacterium* infection, resulting in increased apoptosis of infected cells by the involvement of the TLR2 and NF-κB signaling pathways (71). Downregulation of miR-155 result in decreased TNF-α production in response to lipomannan, a component of the bacterial cell wall, was reported in human macrophages, affecting the TLR-MAPK/Akt signaling pathway (72). In addition, MiR-155 is involved in regulation of autophagy-mediated mycobacterial elimination by the repression of the negative regulator Rheb (73). miR-142-3p participates in an effective strategy in mycobacterial infection to control early events of phagolysosome biogenesis *via* targeting the N-Wasp and actin-binding protein (74). On the other hand, down regulation of miR-142-3p negatively regulates the production of NF-κB, TNF-α, and IL-6 in macrophages upon *Mycobacterium bovis* infection, resulting in the activation of the NF-κB pathway *via* the de-repression of the target IRAK1 (75). miR-124 has been found to serve as a potent modulator of the immune response in an *M. bovis* BCG-infected macrophage cell line (Raw 264.7) by targeting components of the TLR signaling pathway including MyD88, TLR6, TRAF6, and TNF-α (76).

miR-146a modulates the inflammatory response upon *Mycobacterium* infection in Raw 264.7 cells by targeting IRAK1 and TRAF6, resulting in remarkably reduced translation of IL-6, IL-1β, and TNF-α (77). Kumar and colleagues reported that *Mycobacterium tuberculosis* down regulates the expression of miRNA let-7f, which targets mRNA of A20, an inhibitor of NF-κB. Significantly, the downregulation of let-7f is accompanied by concomitant upregulation of A20 in mice infected with *M. tuberculosis* (78).

miR-21 was found to induce inhibition of IL-12 production in a NF-κB-dependent manner in DCs and T-cells upon *Mycobacterium* infection, and thus suppress host Th1 responses (79). Interestingly, miR-21 upregulation promotes DCs apoptosis by targeting Bcl-2 in *Mycobacterium*-infected cells (80). miR-99b upregulation has been observed to stimulate the production of proinflammatory cytokines such as IL-6, IL-12, IL-1β, and TNF-α in macrophages and DCs upon *Mycobacterium* infection. Inhibition of TNF-α production is a key strategy of *Mycobacterium* to promote growth within DCs that, in turn, facilitates evasion of host adaptive immune response mechanisms (81). Transfection of T cells with miR-144 precursor has demonstrated that miR-144 possibly regulates antituberculosis immunity by inhibition of IFN-γ and TNF-α production and T cell proliferation (82). Wang et al. (83) reported that miR-223 and miR-424 promotes monocyte differentiation and subsequently downregulates the expression of transcription factor NFI-A78. The downregulation of miRNAs involved in disorder proportions of T cells and B cells in active tuberculosis patients (83). Collectively, these finding indicates that *Mycobacterium* use miRNA-based strategies for completion of intracellular replication that, in turn, facilitates evasion of the immune response.

### *Brucella* 

**Figure 3:**
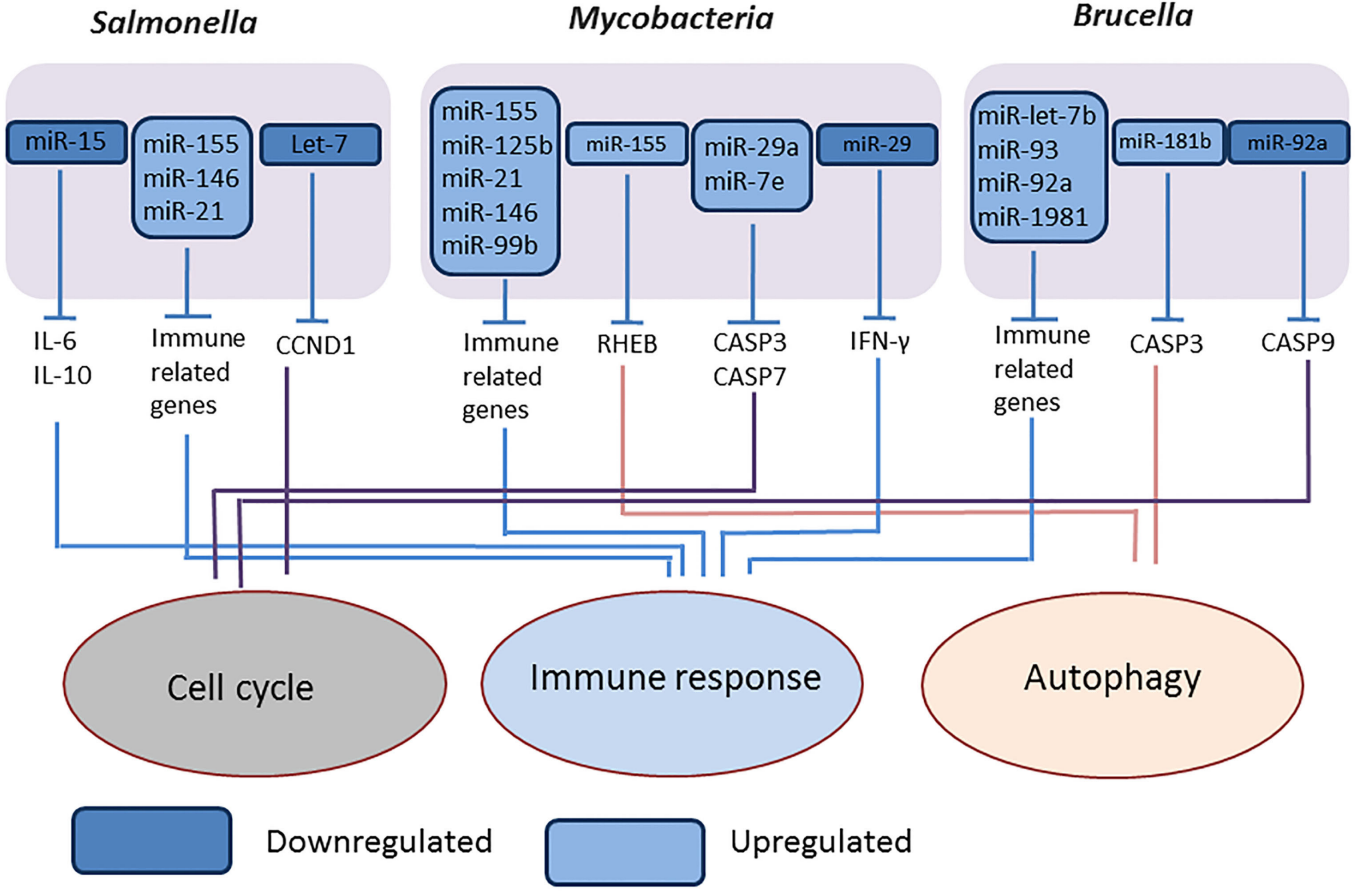
**Overview of modulation of host miRNAs by intracellular bacterial pathogens**. The representative figure of modulation of host miRNAs by intracellular bacterial pathogens, *Salmonella* (Gram negative, intracellular), *Mycobacteria* (intracellular), and *Brucella* (Gram negative, intracellular).

We studied the expression of miRNAs expression in *Brucella melitensis*-infected RAW 264 macrophages cells and found several miRNAs such as miR-let-7b, miR-93, miR-92a, miR-181b, and miR-1981, differentially expressed compared to mock-infected cells, and purposed that these miRNAs are involved in regulation of autophagy, apoptosis, innate and adaptive immune response mechanisms (Figure 3) (84). Liu et al. (85) reported that the downregulation of miR-125b-5p during *B. abortus* infection enhances the expression of the A20 protein, thereby inhibiting NF-κB activation and facilitating bacterial intracellular survival (85).

## Approaches to Identify Bacterial sRNAs

Now that a number of actively transcribed sRNAs have been identified in intracellular bacteria, further research should focus on the determination of their functions and their specific targets. TargetRNA2 was the first webserver specifically designed for identification of bacterial sRNA target which determine sRNA–target interactions through the straight forward hybridization model. It determines the seed region between two putative sRNA targets which is composed of very small short series of consecutive base (86). IntaRNA is also used to identify sRNA targets which work on the principle of hybridization energy between base pairings of sRNA–target interactions to the hybridization energy of interacting regions being unpaired in intramolecular structures. Further, it conform the seed regions more efficiently as compared all other available webtools (87). Target prediction could also be used in conjunction with experimental genome-wide approaches, such as transcriptional profiling (88). Comparison of the transcription profile of a mutant strain or an over-expresser strain to the wild-type strain can highlight putative targets (9). Comparison of the sRNA deletion mutant, the over-expresser strain and the wild-type strain by SDS-PAGE and Coomassie staining may be sufficient to suggest a putative target (89). The proteomic studies could be undertaken or more direct approaches, such as the streptavidin-binding aptamer tag described above could be used (90). Said et al. (91) have performed a systematic analysis for the use of different aptamers and configurations to identify protein targets of sRNA. Further, we need to establish more accurate identification methods for the bacterial sRNA prediction.

## Summary and Perspectives

Recent advancements in research have revealed diverse functions, wide distribution, and high variability of sRNAs and described their crucial role in biological processes, such as infectivity and virulence of intracellular bacteria, stress adaptation, and environmental sensing, as well as in modulation of innate and adaptive immune response mechanisms. Intracellular bacteria are divided in two classes: *vacuolar* intracellular bacteria, such as *Salmonella, Mycobacterium, Legionella, Brucella*, and *Coxiella*, which survive and replicate either by avoiding vacuole–lysosome fusion or by altering the phagolysosome environment; and cytosolic intracellular bacteria, including *Francisella, Shigella, Listeria, Burkholderia*, and *Rickettsia*, which usually escape to proliferate within the cytosol of host cell. Infection is a multidimensional complex event, with host cells having developed immune mechanisms to counteract invading intracellular pathogens *via* lysosomal degradation to maintain a balance between host resistance and bacterial virulence. Intracellular pathogens have evolved several sRNA-based strategies to survive and replicate within phagocytic cells and to manipulate the host-cell machinery for their own benefit. Upon internalization in host cells, the pathogenic bacteria are usually surrounded by a membrane-bound vacuole that protects against proteolytic degradation. Bacterial regulatory sRNAs operate at all levels of gene regulation to modulate translation, transcription, DNA maintenance or silencing, and mRNA stability. They use different mechanisms to perform regulatory functions, including changes in RNA conformation, base pairing with other RNAs, protein binding, and interactions with DNA.

The Csr-type system is the most common posttranscriptional regulator network in intracellular bacteria which participates with type IVB secretion system to modulate the ER–Golgi vesicular trafficking highlighting the involvement of sRNA-binding protein in survival and replication of intracellular pathogens. The effector proteins, VipA, RalF, and YlfA, have been linked directly in vesicular trafficking to affect the host-cell activity. Additionally, sRNA, RsmY and RsmZ, in *Legionella*, RybB sRNA in *Salmonella*, and sRNA AbcR in *Brucella* regulate the expression of effector protein for survival and intracellular replication in macrophages.

At the initial stage of infectious pathogenesis, intracellular pathogens modulate the immune response mechanism of host to quickly translocate through the mucosal immune barrier and are endocytosed by mucosal macrophages and DCs. Intracellular pathogens infect the host-cell machinery by targeting IRAK1 and TRAF6 to limit PRRs which, in turn, affects the TLR/NF-κB signaling cascade. Inhibition of antigen presentation to T cells is a further strategy of intracellular bacteria to dampen innate and adaptive immune mechanisms. They have the ability to hinder activation of DCs to subvert the immune response mechanism by averting function of T-cells and secreting IL-12 to establish a strong Th1 immune response.

Additionally, inhibition of TNF-α production and modulation of MHC-I and MHC-II expression is a key strategy of intracellular pathogen to promote growth within DCs that, in turn, favors cytokine regulation to facilitate evasion of host adaptive immune response mechanisms. Although numerous strategies employed by bacterial sRNAs have been reported, there are many mysteries that are still veiled, including how are sRNAs involved in modulation of innate immune signaling? What is the role of sRNAs in regulation of apoptosis and autophagy mechanisms? To date, nothing is known about the role of sRNA interfering with innate and adaptive immune mechanism of *Brucella* and *Legionella* and it will be an open question for the next few years. In-depth identification of novel immune evasion strategies employed by bacterial sRNAs will facilitate our understanding of pathogenesis and designing of novel effective therapeutic approaches to combat diseases caused by intracellular pathogens.

## Author Contributions

Z-FL and WA conceived the research. WA and KZ wrote the manuscript. Z-FL revised the manuscript critically for relevant intellectual content. All authors have read and approved the manuscript.

## Conflict of Interest Statement

The authors declare that the research was conducted in the absence of any commercial or financial relationships that could be construed as a potential conflict of interest. The reviewer AF and handling editor declared their shared affiliation, and the handling editor states that the process nevertheless met the standards of a fair and objective review.

## References

[1] Fitzgerald KA Caffrey DR. Long noncoding RNAs in innate and adaptive immunity. Curr Opin Immunol (2014) 26:140–6. 10.1016/j.coi.2013.12.001 24556411 PMC3932021

[2] Guttman M Rinn JL. Modular regulatory principles of large non-coding RNAs. Nature (2012) 482:339–46. 10.1038/nature10887 22337053 PMC4197003

[3] Gottesman S The small RNA regulators of *Escherichia coli*: roles and mechanisms. Annu Rev Microbiol (2004) 58:303–28. 10.1146/annurev.micro.58.030603.123841 15487940

[4] Storz G Vogel J Wassarman KM. Regulation by small RNAs in bacteria: expanding frontiers. Mol Cell (2011) 43:880–91. 10.1016/j.molcel.2011.08.022 21925377 PMC3176440

[5] Waters LS Storz G. Regulatory RNAs in bacteria. Cell (2009) 136:615–28. 10.1016/j.cell.2009.01.043 19239884 PMC3132550

[6] Gottesman S Storz G. Bacterial small RNA regulators: versatile roles and rapidly evolving variations. Cold Spring Harb Perspect Biol (2011) 3. 10.1101/cshperspect.a003798 20980440 PMC3225950

[7] Guillier M Gottesman S The 5 end of two redundant sRNAs is involved in the regulation of multiple targets, including their own regulator. Nucleic Acids Res (2008) 36:6781–94. 10.1093/nar/gkn742 18953042 PMC2588501

[8] Frohlich KS Vogel J Activation of gene expression by small RNA. Curr Opin Microbiol (2009) 12:674–82. 10.1016/j.mib.2009.09.009 19880344

[9] Papenfort K Sun Y Miyakoshi M Vanderpool CK Vogel J. Small RNA-mediated activation of sugar phosphatase mRNA regulates glucose homeostasis. Cell (2013) 153:426–37. 10.1016/j.cell.2013.03.003 23582330 PMC4151517

[10] Aiba H. Mechanism of RNA silencing by Hfq-binding small RNAs. Curr Opin Microbiol (2007) 10:134–9. 10.1016/j.mib.2007.03.010 17383928

[11] Johansson J Cossart P. RNA-mediated control of virulence gene expression in bacterial pathogens. Trends Microbiol (2003) 11:280–5. 10.1016/S0966-842X(03)00118-5 12823945

[12] Ebert MS Sharp PA Roles for microRNAs in conferring robustness to biological processes. Cell (2012) 149:515–24. 10.1016/j.cell.2012.04.005 22541426 PMC3351105

[13] Agbor TA McCormick BA. *Salmonella* effectors: important players modulating host cell function during infection. Cell Microbiol (2011) 13:1858–69. 10.1111/j.1462-5822.2011.01701.x 21902796 PMC3381885

[14] Cossart P Sansonetti PJ. Bacterial invasion: the paradigms of enteroinvasive pathogens. Science (2004) 304:242–8. 10.1126/science.1090124 15073367

[15] Hubber A Roy CR. Modulation of host cell function by *Legionella pneumophila* type IV effectors. Annu Rev Cell Dev Biol (2010) 26:261–83. 10.1146/annurev-cellbio-100109-104034 20929312

[16] Fredlund J Enninga J. Cytoplasmic access by intracellular bacterial pathogens. Trends Microbiol (2014) 22:128–37. 10.1016/j.tim.2014.01.003 24530174

[17] Sharma CM Vogel J Experimental approaches for the discovery anal characterization of regulatory small RNA. Curr Opin Microbiol (2009) 12:536–46. 10.1016/j.mib.2009.07.006 19758836

[18] Chao YJ Papenfort K Reinhardt R Sharma CM Vogel J. An atlas of Hfq-bound transcripts reveals 3’UTRs as a genomic reservoir of regulatory small RNAs. EMBO J (2012) 31:4005–19. 10.1038/emboj.2012.229 22922465 PMC3474919

[19] Chao YJ Vogel J. The role of Hfq in bacterial pathogens. Curr Opin Microbiol (2010) 13:24–33. 10.1016/j.mib.2010.01.001 20080057

[20] Saramago M Barria C dos Santos RF Silva IJ Pobre V Domingues S The role of RNases in the regulation of small RNAs. Curr Opin Microbiol (2014) 18:105–15. 10.1016/j.mib.2014.02.009 24704578

[21] Brennan RG Link TM. Hfq structure, function and ligand binding. Curr Opin Microbiol (2007) 10:125–33. 10.1016/j.mib.2007.03.015 17395525

[22] Caldelari I Chao YJ Romby P Vogel J. RNA-mediated regulation in pathogenic bacteria. Cold Spring Harb Perspect Med (2013) 3:a010298. 10.1101/cshperspect.a010298 24003243 PMC3753719

[23] Wagner EGH. Cycling of RNAs on Hfq. RNA Biol (2013) 10:619–26. 10.4161/rna.24044 23466677 PMC3710369

[24] Duss O Michel E Konte NDD Schubert M Allain FHT. Molecular basis for the wide range of affinity found in Csr/Rsm protein-RNA recognition. Nucleic Acids Res (2014) 42:5332–46. 10.1093/nar/gku141 24561806 PMC4005645

[25] Jinek M Jiang FG Taylor DW Sternberg SH Kaya E Ma EB Structures of Cas9 endonucleases reveal RNA-mediated conformational activation. Science (2014) 343:1247997. 10.1126/science.1247997 24505130 PMC4184034

[26] Stern A Keren L Wurtzel O Amitai G Sorek R. Self-targeting by CRISPR: gene regulation or autoimmunity? Trends Genet (2010) 26:335–40. 10.1016/j.tig.2010.05.008 20598393 PMC2910793

[27] Alix E Mukherjee S Roy CR Host-pathogen interactions subversion of membrane transport pathways by vacuolar pathogens. J Cell Biol (2011) 195:943–52. 10.1083/jcb.201105019 22123831 PMC3241728

[28] Ahmed W Zheng K Liu ZF. Establishment of chronic infection: *Brucella*’s stealth strategy. Front Cell Infect Microbiol (2016) 6:30. 10.3389/fcimb.2016.00030 27014640 PMC4791395

[29] Faucher SP Mueller CA Shuman HA. *Legionella pneumophila* transcriptome during intracellular multiplication in human macrophages. Front Microbiol (2011) 2:60. 10.3389/fmicb.2011.00060 21747786 PMC3128937

[30] Ortega AD Quereda JJ Pucciarelli MG Garcia-del Portillo F Non-coding RNA regulation in pathogenic bacteria located inside eukaryotic cells. Front Cell Infect Microbiol (2014) 4:162 10.3389/fcimb.2014.00162 25429360 PMC4228915

[31] Faucher SP Friedlander G Livny J Margalit H Shuman HA. *Legionella pneumophila* 6S RNA optimizes intracellular multiplication. Proc Natl Acad Sci U S A (2010) 107:7533–8. 10.1073/pnas.0911764107 20368425 PMC2867745

[32] Nevo O Zusman T Rasis M Lifshitz Z Segal G. Identification of *Legionella pneumophila* effectors regulated by the LetAS-RsmYZ-CsrA regulatory cascade, many of which modulate vesicular trafficking. J Bacteriol (2014) 196:681–92. 10.1128/JB.01175-13 24272784 PMC3911145

[33] Heidtman M Chen EJ Moy MY Isberg RR. Large-scale identification of *Legionella pneumophila* Dot/Icm substrates that modulate host cell vesicle trafficking pathways. Cell Microbiol (2009) 11:230–48. 10.1111/j.1462-5822.2008.01249.x 19016775 PMC2744955

[34] Jonas K Edwards AN Ahmad I Romeo T Romling U Melefors O. Complex regulatory network encompassing the Csr, c-di-GMP and motility systems of *Salmonella* Typhimurium. Environ Microbiol (2010) 12:524–40. 10.1111/j.1462-2920.2009.02097.x 19919539 PMC2888478

[35] Forsbach-Birk V McNealy T Shi C Lynch D Marre R. Reduced expression of the global regulator protein CsrA in *Legionella pneumophila* affects virulence-associated regulators and growth in *Acanthamoeba castellanii*. Int J Med Microbiol (2004) 294:15–25. 10.1016/j.ijmm.2003.12.003 15293450

[36] Martinez LC Yakhnin H Camacho MI Georgellis D Babitzke P Puente JL Integration of a complex regulatory cascade involving the SirA/BarA and Csr global regulatory systems that controls expression of the *Salmonella* SPI-1 and SPI-2 virulence regulons through HilD. Mol Microbiol (2011) 80:1637–56. 10.1111/j.1365-2958.2011.07674.x 21518393 PMC3116662

[37] Kulkarni PR Jia T Kuehne SA Kerkering TM Morris ER Searle MS A sequence-based approach for prediction of CsrA/RsmA targets in bacteria with experimental validation in *Pseudomonas aeruginosa*. Nucleic Acids Res (2014) 42:6811–25. 10.1093/nar/gku309 24782516 PMC4066749

[38] Rasis M Segal G The LetA-RsmYZ-CsrA regulatory cascade, together with RpoS and PmrA, post-transcriptionally regulates stationary phase activation of *Legionella pneumophila* Icm/Dot effectors. Mol Microbiol (2009) 72:995–1010. 10.1111/j.1365-2958.2009.06705.x 19400807

[39] Jayakumar D Early JV Steinman HM. Virulence phenotypes of *Legionella pneumophila* associated with noncoding RNA lpr0035. Infect Immun (2012) 80:4143–53. 10.1128/IAI.00598-12 22966048 PMC3497406

[40] Trigui H Mendis N Li L Saad M Faucher SP Facets of small RNA-mediated regulation in *Legionella pneumophila*. Molecular Mechanisms in Legionella Pathogenesis. (Vol. 376) (2014). p. 53–80. 10.1007/82_2013_347 23918178

[41] Leclerc JM Dozois CM Daigle F. Role of the *Salmonella enterica* serovar Typhi Fur regulator and small RNAs RfrA and RfrB in iron homeostasis and interaction with host cells. Microbiology (2013) 159:591–602. 10.1099/mic.0.064329-0 23306672

[42] Ortega AD Gonzalo-Asensio J Garcia-del Portillo F. Dynamics of *Salmonella* small RNA expression in non-growing bacteria located inside eukaryotic cells. RNA Biol (2012) 9:469–88. 10.4161/rna.19317 22336761

[43] Caswell CC Gaines JM Ciborowski P Smith D Borchers CH Roux CM Identification of two small regulatory RNAs linked to virulence in *Brucella abortus* 2308. Mol Microbiol (2012) 85:345–60. 10.1111/j.1365-2958.2012.08117.x 22690807 PMC3391331

[44] Caswell CC Gaines JM Roop RM The RNA chaperone Hfq independently coordinates expression of the VirB type IV secretion system and the LuxR-type regulator BabR in *Brucella abortus* 2308. J Bacteriol (2012) 194:3–14. 10.1128/Jb.05623-11 22020650 PMC3256608

[45] McNealy TL Forsbach-Birk V Shi CW Marre R. The Hfq homolog in *Legionella pneumophila* demonstrates regulation by LetA and RpoS and interacts with the global regulator CsrA. J Bacteriol (2005) 187:1527–32. 10.1128/JB.187.4.1527-1532.2005 15687220 PMC545622

[46] Sittka A Lucchini S Papenfort K Sharma CM Rolle K Binnewies TT Deep sequencing analysis of small noncoding RNA and mRNA targets of the global post-transcriptional regulator, Hfq. PLoS Genet (2008) 4:e1000163. 10.1371/journal.pgen.1000163 18725932 PMC2515195

[47] Sittka A Pfeiffer V Tedin K Vogel J. The RNA chaperone Hfq is essential for the virulence of *Salmonella typhimurium*. Mol Microbiol (2007) 63:193–217. 10.1111/j.1365-2958.2006.05489.x 17163975 PMC1810395

[48] Ansong C Yoon H Porwollik S Mottaz-Brewer H Petritis BO Jaitly N Global systems-level analysis of Hfq and SmpB deletion mutants in *Salmonella*: implications for virulence and global protein translation. PLoS One (2009) 4(3):e4809. 10.1371/journal.pone.0004809 19277208 PMC2652828

[49] Sharma CM Papenfort K Pernitzsch SR Mollenkopf HJ Hinton JCD Vogel J. Pervasive post-transcriptional control of genes involved in amino acid metabolism by the Hfq-dependent GcvB small RNA. Mol Microbiol (2011) 81:1144–65. 10.1111/j.1365-2958.2011.07751.x 21696468

[50] Gong H Vu GP Bai Y Chan E Wu RB Yang E A *Salmonella* small non-coding RNA facilitates bacterial invasion and intracellular replication by modulating the expression of virulence factors. PLoS Pathog (2011) 7:e1002120. 10.1371/journal.ppat.1002120 21949647 PMC3174252

[51] Padalon-Brauch G Hershberg R Elgrably-Weiss M Baruch K Rosenshine I Margalit H Small RNAs encoded within genetic islands of *Salmonella typhimurium* show host-induced expression and role in virulence. Nucleic Acids Res (2008) 36:1913–27. 10.1093/nar/gkn050 18267966 PMC2330248

[52] Lee EJ Groisman EA. An antisense RNA that governs the expression kinetics of a multifunctional virulence gene. Mol Microbiol (2010) 76:1020–33. 10.1111/j.1365-2958.2010.07161.x 20398218 PMC2909850

[53] DiChiara JM Contreras-Martinez LM Livny J Smith D McDonough KA Belfort M. Multiple small RNAs identified in *Mycobacterium bovis* BCG are also expressed in *Mycobacterium tuberculosis* and *Mycobacterium smegmatis*. Nucleic Acids Res (2010) 38:4067–78. 10.1093/nar/gkq101 20181675 PMC2896511

[54] Diacovich L Gorvel JP. Bacterial manipulation of innate immunity to promote infection. Nat Rev Microbiol (2010) 8:117–28. 10.1038/nrmicro2295 20075926

[55] Voinnet O Micro-balancing innate immunity to *Salmonella*. EMBO J (2011) 30:1877–9. 10.1038/emboj.2011.134 21593727 PMC3098492

[56] Schulte LN Westermann AJ Vogel J. Differential activation and functional specialization of miR-146 and miR-155 in innate immune sensing. Nucleic Acids Res (2013) 41:542–53. 10.1093/nar/gks1030 23143100 PMC3592429

[57] Schulte LN Eulalio A Mollenkopf HJ Reinhardt R Vogel J. Analysis of the host microRNA response to *Salmonella* uncovers the control of major cytokines by the let-7 family. EMBO J (2011) 30:1977–89. 10.1038/emboj.2011.94 21468030 PMC3098495

[58] Sharbati S Sharbati J Hoeke L Bohmer M Einspanier R. Quantification and accurate normalisation of small RNAs through new custom RT-qPCR arrays demonstrates *Salmonella*-induced microRNAs in human monocytes. BMC Genomics (2012) 13:23. 10.1186/1471-2164-13-23 22248082 PMC3268085

[59] Hoeke L Sharbati J Pawar K Keller A Einspanier R Sharbati S. Intestinal *Salmonella typhimurium* infection leads to miR-29a induced caveolin 2 regulation. PLoS One (2013) 8:e67300. 10.1371/journal.pone.0067300 23826261 PMC3691122

[60] Rodriguez A Vigorito E Clare S Warren MV Couttet P Soond DR Requirement of bic/microRNA-155 for normal immune function. Science (2007) 316:608–11. 10.1126/science.1139253 17463290 PMC2610435

[61] Ordas A Kanwal Z Lindenberg V Rougeot J Mink M Spaink HP microRNA-146 function in the innate immune transcriptome response of zebrafish embryos to *Salmonella typhimurium* infection. BMC Genomics (2013) 14:696. 10.1186/1471-2164-14-696 24112639 PMC3852110

[62] Taganov KD Boldin MP Chang KJ Baltimore D. NF-kappa B-dependent induction of microRNA miR-146, an inhibitor targeted to signaling proteins of innate immune responses. Proc Natl Acad Sci U S A (2006) 103:12481–6. 10.1073/pnas.0605298103 16885212 PMC1567904

[63] Zhang TF Yu JX Zhang YQ Li LM Chen YY Li DH *Salmonella enterica* serovar Enteritidis modulates intestinal epithelial miR-128 levels to decrease macrophage recruitment via macrophage colony-stimulating factor. J Infect Dis (2014) 209:2000–11. 10.1093/infdis/jiu006 24415783 PMC4481728

[64] Curtale G Citarella F. Dynamic nature of noncoding RNA regulation of adaptive immune response. Int J Mol Sci (2013) 14:17347–77. 10.3390/ijms140917347 23975170 PMC3794731

[65] Mastroeni P Grant A Restif O Maskell D. A dynamic view of the spread and intracellular distribution of *Salmonella enterica*. Nat Rev Microbiol (2009) 7:73–80. 10.1038/nrmicro2034 19079353

[66] Riquelme SA Bueno SM Kalergis AM. IgG keeps virulent *Salmonella* from evading dendritic cell uptake. Immunology (2012) 136:291–305. 10.1111/j.1365-2567.2012.03578.x 22352313 PMC3385029

[67] Vigorito E Perks KL Abreu-Goodger C Bunting S Xiang Z Kohlhaas S microRNA-155 regulates the generation of immunoglobulin class-switched plasma cells. Immunity (2007) 27:847–59. 10.1016/j.immuni.2007.10.009 18055230 PMC4135426

[68] Maudet C Mano M Eulalio A. microRNAs in the interaction between host and bacterial pathogens. FEBS Lett (2014) 588:4140–7. 10.1016/j.febslet.2014.08.002 25128459

[69] Bao H Kommadath A Liang GX Sun X Arantes AS Tuggle CK Genome-wide whole blood microRNAome and transcriptome analyses reveal miRNA-mRNA regulated host response to foodborne pathogen *Salmonella* infection in swine. Sci Rep (2015) 5:12620. 10.1038/srep12620 26227241 PMC4521145

[70] Harapan H Fitra F Ichsan I Mulyadi M Miotto P Hasan NA The roles of microRNAs on tuberculosis infection: meaning or myth? Tuberculosis (Edinb) (2013) 93:596–605. 10.1016/j.tube.2013.08.004 24025365 PMC4889429

[71] Ghorpade DS Leyland R Kurowska-Stolarska M Patil SA Balaji KN. microRNA-155 is required for *Mycobacterium bovis* BCG-mediated apoptosis of macrophages. Mol Cell Biol (2012) 32:2239–53. 10.1128/MCB.06597-11 22473996 PMC3372268

[72] Rajaram MVS Ni B Morris JD Brooks MN Carlson TK Bakthavachalu B *Mycobacterium tuberculosis* lipomannan blocks TNF biosynthesis by regulating macrophage MAPK-activated protein kinase 2 (MK2) and microRNA miR-125b. Proc Natl Acad Sci U S A (2011) 108:17408–13. 10.1073/pnas.1112660108 21969554 PMC3198317

[73] Wang JL Yang K Zhou L Minhaowu Wu YJ Zhu M microRNA-155 promotes autophagy to eliminate intracellular mycobacteria by targeting Rheb. PLoS Pathog (2013) 9:e1003697. 10.1371/journal.ppat.1003697 24130493 PMC3795043

[74] Bettencourt P Marion S Pires D Santos LF Lastrucci C Carmo N Actin-binding protein regulation by microRNAs as a novel microbial strategy to modulate phagocytosis by host cells: the case of N-Wasp and miR-142-3p. Front Cell Infect Microbiol (2013) 3:19. 10.3389/fcimb.2013.00019 23760605 PMC3672780

[75] Zhang Y Li YK. microRNAs in the regulation of immune response against infections. J Zhejiang Univ Sci B (2013) 14:1–7. 10.1631/jzus.B1200292 23303626 PMC3542953

[76] Ma F Xu S Liu XG Zhang Q Xu XF Liu MF The microRNA miR-29 controls innate and adaptive immune responses to intracellular bacterial infection by targeting interferon-gamma. Nat Immunol (2011) 12:861–U865. 10.1038/ni.2073 21785411

[77] Li S Yue Y Xu W Xiong SD. microRNA-146a represses mycobacteria-induced inflammatory response and facilitates bacterial replication via targeting IRAK-1 and TRAF-6. PLoS One (2013) 8:e81438. 10.1371/journal.pone.0081438 24358114 PMC3864784

[78] Kumar M Sahu SK Kumar R Subuddhi A Maji RK Jana K microRNA let-7 modulates the immune response to *Mycobacterium tuberculosis* infection via control of A20, an inhibitor of the NF-kappa B pathway. Cell Host Microbe (2015) 17:345–56. 10.1016/j.chom.2015.01.007 25683052

[79] Wu ZW Lu HF Sheng JF Li LJ. Inductive microRNA-21 impairs anti-mycobacterial responses by targeting IL-12 and Bcl-2. FEBS Lett (2012) 586:2459–67. 10.1016/j.febslet.2012.06.004 22710123

[80] Riendeau CJ Kornfeld H. THP-1 cell apoptosis in response to mycobacterial infection. Infect Immun (2003) 71:254–9. 10.1128/IAI.71.1.254-259.2003 12496173 PMC143334

[81] Singh Y Kaul V Mehra A Chatterjee S Tousif S Dwivedi VP *Mycobacterium tuberculosis* controls microRNA-99b (miR-99b) expression in infected murine dendritic cells to modulate host immunity. J Biol Chem (2013) 288:5056–61. 10.1074/jbc.C112.439778 23233675 PMC3576108

[82] Liu YH Wang XJ Jiang J Cao ZH Yang BF Cheng XX. Modulation of T cell cytokine production by miR-144* with elevated expression in patients with pulmonary tuberculosis. Mol Immunol (2011) 48:1084–90. 10.1016/j.molimm.2011.02.001 21367459

[83] Wang C Yang SY Sun G Tang XY Lu SH Neyrolles O Comparative miRNA expression profiles in individuals with latent and active tuberculosis. PLoS One (2011) 6:e25832. 10.1371/journal.pone.0025832 22003408 PMC3189221

[84] Zheng K Chen DS Wu YQ Xu XJ Zhang H Chen CF microRNA expression profile in RAW264.7 cells in response to *Brucella melitensis* infection. Int J Biol Sci (2012) 8:1013–22. 10.7150/ijbs.3836 22904669 PMC3421232

[85] Liu N Wang L Sun C Yang L Sun W Peng Q. microRNA-125b-5p suppresses *Brucella abortus* intracellular survival via control of A20 expression. BMC Microbiol (2016) 16:171. 10.1186/s12866-016-0788-2 27473222 PMC4966734

[86] Tjaden B Goodwin SS Opdyke JA Guillier M Fu DX Gottesman S Target prediction for small, noncoding RNAs in bacteria. Nucleic Acids Res (2006) 34:2791–802. 10.1093/nar/gkl356 16717284 PMC1464411

[87] Wright PR Georg J Mann M Sorescu DA Richter AS Lott S CopraRNA and IntaRNA: predicting small RNA targets, networks and interaction domains. Nucleic Acids Res (2014) 42:W119–23. 10.1093/nar/gku359 24838564 PMC4086077

[88] Modi SR Camacho DM Kohanski MA Walker GC Collins JJ. Functional characterization of bacterial sRNAs using a network biology approach. Proc Natl Acad Sci U S A (2011) 108:15522–7. 10.1073/pnas.1104318108 21876160 PMC3174649

[89] Urban JH Vogel J Translational control and target recognition by *Escherichia coli* small RNAs in vivo. Nucleic Acids Res (2007) 35:1018–37. 10.1093/nar/gkl1040 17264113 PMC1807950

[90] Wang J Rennie W Liu C Carmack CS Prevost K Caron MP Identification of bacterial sRNA regulatory targets using ribosome profiling. Nucleic Acids Res (2015) 43:10308–20. 10.1093/nar/gkv1158 26546513 PMC4666370

[91] Said N Rieder R Hurwitz R Deckert J Urlaub H Vogel J. In vivo expression and purification of aptamer-tagged small RNA regulators. Nucleic Acids Res (2009) 37:e133. 10.1093/nar/gkp719 19726584 PMC2777422

